# Vancomycin-resistant Enterococci, Mexico City

**DOI:** 10.3201/eid1305.061421

**Published:** 2007-05

**Authors:** Jennifer Cuellar-Rodríguez, Arturo Galindo-Fraga, Víctor Guevara, Carolina Pérez-Jiménez, Luis Espinosa-Aguilar, Ana Lilia Rolón, Araceli Hernández-Cruz, Esaú López-Jácome, Miriam Bobadilla-del-Valle, Areli Martínez-Gamboa, Alfredo Ponce-de-León, José Sifuentes-Osornio

**Affiliations:** * Instituto Nacional de Ciencias Médicas y Nutrición Salvador Zubirán, Mexico City, Mexico

**Keywords:** Vancomycin-resistant enterococci, Mexico, VRE, antimicrobial resistance, outbreak, letter

**To the Editor:** Vancomycin-resistant *Enterococcus* (VRE) has become an important nosocomial pathogen because of its rapid spread, limited therapy options, mortality, and the possibility of transfer of vancomycin resistance to other pathogens such as *Staphylococcus aureus*. Vancomycin-resistant *E. faecium* (VREF) and *E. faecalis* were first described in 1988 (*1*,*2*).They have become major nosocomial pathogens, but their prevalence in Latin America has remained <2% (*3*). In Mexico, VRE has rarely been reported (*4*,*5*). In a recent study in Mexico City, 100% (n = 60) of the isolates of *E. faecium* and *E. faecalis* were susceptible to vancomycin (*6*).

From May 2004 to April 2005, the rate of vancomycin resistance among all *Enterococcus* isolates was 0.27%. However, in May 2005 the first fully VREF was isolated at our hospital, and the rate of vancomycin resistance was 6.23% (a 23-fold increase) during the following 12-month period.

We performed a retrospective study to describe the isolates and the characteristics of patients with VREF. All VREF isolates from May 2005 through April 2006 were included. We collected demographic and clinical data. For the final identification of the isolates, the VITEK system (bioMérieux, Lyon, France) with VITEK GPI cards (bioMérieux, Inc., Durham NC, USA) were used. Antimicrobial drug susceptibility was tested by using the VITEK GPS-111 card and confirmed by MIC determination that used broth microdilution. Resistance to vancomycin and teicoplanin was confirmed by E-test (AB Biodisk, Solna, Sweden). An isolate was considered vancomycin resistant when the MIC was ≥32 μg/mL and was considered to have high-level resistance when the MIC was ≥256 μg/mL. A PCR for detection of the *vanA* or *vanB* genotype was used (*7*). Isolates were characterized by pulsed-field gel electrophoresis (PFGE) (*8*,*9*); a dendrogram was constructed with the GelCompare II 4.0 software (Applied Maths, Kortrijk, Belgium), and the similarity was compared with the Dice coefficient.

In the study period, VREF was isolated from 27 patients. The median age was 40 years (range 22–84 years). VREF was isolated from the abdomen in 14 patients (51.9%); 11 isolates were from an abscess, 2 from infected surgical sites, and 1 from ascites. An additional 8 isolates were from the urinary tract (29.6%), 2 from the bloodstream (7.4%), 2 from soft-tissue (7.4%), and 1 (3.7%) from bone. Residence in the general medical wards during the isolation of VREF was most common, 17 (63%) cases, followed by 6 (22.2%) in the intensive care unit. The remaining 4 (14.8%) were distributed in other areas. Median time of hospitalization before the isolation was 21 days (range 1–84 days). Twenty-five patients (92.6%) had a central line, 12 (44.4%) had mechanical ventilation, and 20 (74.1%) previous surgery. Of the last group, 17 (85%) of 20 had abdominal surgery. Twenty-four patients (88.8%) received an antimicrobial drug before the isolation of VREF: third- or fourth-generation cephalosporins (89%), metronidazole (70.4%), aminoglycosides (70.4%), vancomycin (66.7%), carbapenems (66.7%), amoxicillin or ampicillin (48.1%), antifungal agents (48.1%); and <20% received quinolones, trimethoprim-sulfamethoxazole, colistin, macrolides, and antimycobacterial or antiviral agents. The median time of antimicrobial drug use was 11 days (range 1–84 days). During hospitalization, 7 patients died (crude death rate, 25.9%), 5 of them from sepsis with at least another microorganism isolated; the remaining 2 died of gastrointestinal hemorrhage.

All isolates of *E. faecium* had a vancomycin MIC ≥256 μg/mL and a vanA phenotype (teicoplanin resistance); 26 (96.3%) had *vanA* genotype. Only 1 isolate of *E. faecium* was classified as non–*vanA,* non *vanB,* even though it demonstrated high-level resistance to vancomycin and teicoplanin. Resistance to other antimicrobial agents was as follows: ampicillin and ciprofloxacin, 100%; high-level gentamicin, 48.2%; quinupristin/dalfopristin, 7.4%; and linezolid, 0%.

PFGE analysis showed several genotypes of *E. faecium*; however, 18 of 26 of the isolates had <3 band differences from the predominant strain classified as type A. One isolate of *E. faecium* could not be typed (Figure).

**Figure:**
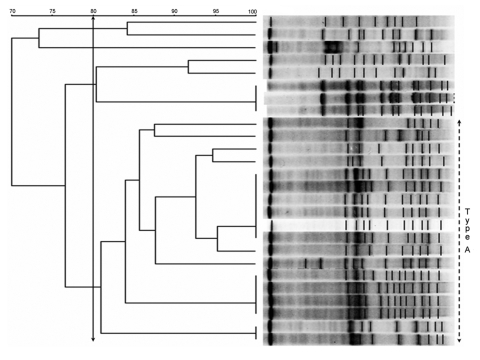
Pulsed-field gel electrophoresis (PFGE) banding patterns of chromosomal DNA of 26 isolates of vancomycin-resistant enterococci. There is a clear predominant type, classified as type A (≥80% similarity), composed of 18 isolates of *Enterococcus faecium.* There are at least 3 subtypes that display a 100% similarity.

As in most tertiary-care centers, our PFGE data suggest that a heterogenous population of VREF exists, but a particular clone established itself as the dominant strain. Although infection control measures are well established in our hospital, in disseminated outbreaks caused by several different clones, infection control measures and control of vancomycin use have shown only limited efficacy. This suggests selection pressure by antimicrobial drugs other than vancomycin (*10*). Early detection of VREF is of extreme importance because of the possibility that the *vanA* gene may be transferred to a variety of gram-positive microorganisms, including *S. aureus.*

The rate of isolation of VREF at our hospital increased considerably during the last year. Even though the number of patients is small, we consider this finding to be of utmost importance, since VREF seems to be emerging in Mexico. To our knowledge, this is the first well-documented outbreak of high-level resistance to vancomycin in enterococci in Mexico. Further research is needed to determine if the problem is limited to our hospital or if it is a nationwide trend.
